# Urethral Strictures After Endoscopic Enucleation of the Prostate and Its Associated Clinical Outcomes in Aging Men

**DOI:** 10.3390/medicina60111771

**Published:** 2024-10-29

**Authors:** Chen-Pang Hou, Jen-Hsuan Wu, Shu-Chuan Weng, Yu-Hsiang Lin, Chien-Lun Chen, Han-Yu Tsai, Yu-Ting Chen, Horng-Heng Juang

**Affiliations:** 1 Department of Urology, Chang Gung Memorial Hospital at Linkou, Taoyuan 333, Taiwan; sss13132002@cgmh.org.tw (J.-H.W.); linyh@cgmh.org.tw (Y.-H.L.); clc2679@cgmh.org.tw (C.-L.C.); b9802087@cgmh.org.tw (H.-Y.T.); tim1452@cgmh.org.tw (Y.-T.C.); 2 Department of Healthcare Management, Yuanpei University of Medical Technology, Hsinchu 330, Taiwan; scweng303@mail.ypu.edu.tw; 3 School of Medicine, Chang Gung University, Taoyuan 333, Taiwan; hhj143@mail.cgu.edu.tw

**Keywords:** benign prostatic hyperplasia, endoscopic enucleation, urethral strictures, urinary tract Infection

## Abstract

*Background and Objectives:* Benign prostatic hyperplasia is a common condition among aging men, leading to bladder outlet obstruction and associated lower urinary tract symptoms. Surgical intervention, particularly endoscopic enucleation of the prostate, has become increasingly popular over traditional methods such as transurethral resection of the prostate. However, urethral strictures remain a major postoperative complication. This study evaluated the incidence, risk factors, and clinical outcomes of urethral strictures after endoscopic enucleation of the prostate. *Materials and Methods:* This study retrospectively analyzed prospectively collected data from 246 patients who underwent either thulium laser enucleation of the prostate or bipolar transurethral enucleation of the prostate at Chang Gung Memorial Hospital between October 2018 and December 2022. Patients were evaluated preoperatively using uroflowmetry, International Prostate Symptom Score (IPSS), and other relevant clinical metrics. Follow-up assessments at 2 weeks, 3 months, and 6 months post-surgery included uroflowmetry, IPSS evaluation, and cystoscopy when indicated. A urethral stricture was deemed to be present if a 5.5 mm fiber cystoscope was unable to pass through the urethra. *Results:* Of the 246 patients, 23 (9.3%) developed urethral strictures, with the membranous urethra being the most common site (69.2%). Patients with strictures had significantly smaller prostate volumes and enucleated tissue weights, higher trial without catheter (TWOC) failure rates, and a higher postoperative urinary tract infection (UTI) incidence. Multivariate analysis identified smaller prostate size, lower resected tissue weight, TWOC failure, and postoperative UTI as significant risk factors for stricture formation. The type of energy source used for enucleation (bipolar or Thulium laser) was not identified as a factor influencing the incidence of urethral stricture. *Conclusions:* Urethral strictures constitute a major complication following endoscopic enucleation of the prostate, particularly in patients with smaller prostates and those experiencing postoperative complications such as UTIs and TWOC failure. These findings underscore the importance of careful surgical technique and rigorous postoperative monitoring to reduce the incidence of this complication.

## 1. Introduction

Benign prostatic hyperplasia (BPH) is a prevalent and likely unavoidable condition in aging men. The incidence of BPH increases with age, with the disease affecting nearly 60% of men by the age of 65 [1]. BPH is characterized by the gradual, benign enlargement of the prostate gland owing to the uncontrolled hyperplastic growth of epithelial and fibromuscular tissues in the transition zone and periurethral area, leading to bladder outlet obstruction [1]. Bladder outlet obstruction is clinically associated with lower urinary tract symptoms, which traditionally include storage symptoms (e.g., frequency, urgency, nocturia, and incontinence) and voiding symptoms (e.g., weak stream and urinary retention). The initial treatment for this condition typically involves lifestyle changes and medications [2]. Surgical treatment is advantageous for patients with BPH who experience urinary retention, renal insufficiency, chronic urinary tract infections (UTIs), involvement of diverticula, recurrent gross hematuria, and bladder stones, as well as for those who do not respond to medical therapy [2]. Transurethral resection of the prostate (TURP) is the most performed surgical treatment for BPH [3]. Common complications of TURP include acute postoperative urinary retention necessitating recatheterization, transient incontinence, hematuria necessitating additional surgery, bladder neck contracture, urethral strictures, and UTIs [4]. Among these complications, urethral strictures are relatively frequent and challenging to manage for both clinicians and patients, often necessitating another surgical intervention. The literature indicates that urethral strictures occur in 4.5% to 13% of patients undergoing transurethral prostate surgery, and the membranous urethra is the most common location for these strictures [5].

In recent years, numerous emerging transurethral prostate surgery techniques, particularly transurethral enucleation of the prostate, have been gradually replacing the traditional TURP technique. The body of research on enucleation as a treatment for BPH has been growing since 1988, particularly between 2007 and 2018 [6]. Bipolar transurethral enucleation of the prostate (B-TUEP), thulium laser enucleation of the prostate, and holmium laser enucleation of the prostate have increasingly become commonly used alternatives to the traditional TURP technique. These new techniques have demonstrated numerous advantages in terms of surgical outcomes and safety [7,8]. However, research on urethral strictures caused by endoscopic enucleation of the prostate is limited. In contrast to traditional techniques that involve the use of heat conduction from electrocautery loops to resect prostate tissue or laser energy to vaporize it, endoscopic enucleation of the prostate focuses more on using physical force to separate the prostate adenoma from the capsule [9]. Accordingly, we believe that this topic warrants more in-depth investigation, and we designed this study to investigate the relationship between urethral stricture and endoscopic prostate enucleation.

## 2. Materials and Methods

### 2.1. Patient Selection and Evaluation

This study retrospectively reviewed data that had been prospectively collected from selected patients who had symptomatic BPH and underwent either 120 W thulium/yttrium–aluminum–garnet laser enucleation of the prostate or B-TUEP at the Geriatric Urology Department of Chang Gung Memorial Hospital in Taiwan. The study was conducted from October 2018 to December 2022 and was approved by the Institutional Review Board (IRB) of Chang Gung Memorial Hospital (IRB number: 202201149B0). All included patients had been receiving medical treatment for BPH for at least 3 months prior to their surgery and met the surgical criteria for bladder outlet obstruction [10]. All surgeries were performed by a single experienced surgeon. Uroflowmetry was used to evaluate voiding ability, including voiding volume, peak flow rate (Qmax), uroflow pattern, and postvoid residual (PVR). Additionally, each patient’s International Prostate Symptom Score (IPSS) and IPSS quality of life score were documented. Patients were included in the study if they had a prostate volume of >30 cm^3^, an IPSS of >20, a Qmax value of <15 mL/s, and an Eastern Cooperative Oncology Group performance status score of <2 [11]. Data on all preoperative variables were derived from the most recent examination conducted prior to the surgical procedure. To exclude patients with prostate cancer, a transrectal ultrasound-guided biopsy was performed when prostate cancer was suspected, which was indicated by abnormal digital rectal examination findings, prostate-specific antigen levels of >4 ng/mL, or the presence of a hypoechoic lesion in transrectal ultrasound-guided biopsy images. Our standard protocol for preoperative urine testing and antibiotic administration involved the following steps: A urinalysis and urine culture were performed one day prior to surgery to identify any infections. If bacteriuria was detected, targeted antibiotic therapy was initiated based on culture sensitivity results. In the absence of bacteriuria or pyuria, a single dose of antibiotics was administered within 1 h before the procedure to prevent perioperative infections. Postoperatively, patients received oral antibiotic treatment for 3 days. Patients with symptomatic UTI were avoided from surgery, as their infection had to be fully treated before they became eligible for the procedure. This study also excluded patients with a history of prostate surgery or active malignancy and those with neurogenic bladder or lower urinary tract symptoms unrelated to BPH.

### 2.2. Surgical Equipment and Procedures

All laser enucleation procedures were performed using a 120 W thulium laser (Vela XL, Boston Scientific, Marlborough, MA, USA) with a continuous wavelength of 1.94 μm. A continuous flow 26 Ch. OES-Pro resectoscope (Olympus Europe, Hamburg, Germany), equipped with a 12° optic and a dedicated operative channel for the fiber, was used. The surgical technique followed the procedure outlined by Herrmann et al. [12]. The prostatic adenoma within the bladder lumen was fragmented using a mechanical tissue morcellator (Piranha©, Richard Wolf GmbH, Knittlingen, Germany), which was guided through a long nephoscope connected to the outer sheath of a resectoscope using an adapter (Karl Storz GmbH, Tuttlingen, Germany). Patients who underwent B-TUEP were treated using an Olympus SurgMaster UES-40 bipolar generator and a 26 Fr. OES-Pro bipolar resectoscope (Olympus Europe, Hamburg, Germany). The surgical technique followed the procedure outlined by Liu et al. [13]. Antibiotics were administered both before and after surgery, in accordance with the recommended protocol [14].

### 2.3. Postoperative Follow-Up

**Figure 1:**
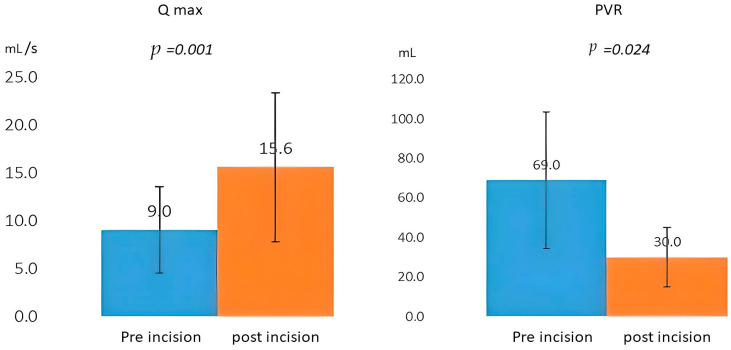
The Qmax and PVR results obtained before and after the incision.

Patients attended follow-up appointments at 2 weeks, 3 months, and 6 months after discharge. During these visits, we evaluated their IPSS values, quality of life scores, Qmax values, voiding volumes, uroflow patterns, and PVR levels. For patients who underwent enucleation of the prostate, cystoscopy was proactively performed if they reported worsening urinary symptoms—which was confirmed by deteriorating urodynamic test results—after the possibility of infections had been excluded (Figure 1). A urethral stricture was deemed to be present if the Olympus CYF-5 fiber cystoscope (outer diameter 5.5 mm) was unable to reach the bladder. The location of the urethral stricture was documented during cystoscopy. Patients were considered to have postoperative UTI if they reported difficulties in urinating, frequent urination, or urethral pain within a month after surgery; showed pyuria in urine analysis; and exhibited improvement in symptoms after antibiotic treatment.

### 2.4. Statistical Analysis

Continuous variables were assessed using an independent-samples *t*-test, and categorical variables were assessed using a chi-square test. Binary logistic regression was used for univariate and multivariate analyses to identify independent factors affecting the outcomes of interest. All variables included in our analysis were tested for multicollinearity. *p * <  0.05 was considered to indicate statistical significance. The analyses were performed using SPSS (version 25.0; IBM).

## 3. Results

We reviewed the data of 263 patients, of whom 17 were excluded from the analysis; 16 were found to have prostate cancer, and 1 was found to have concomitant urothelial carcinoma. Therefore, a total of 246 patients were enrolled in the study, of whom 110 underwent B-TUEP and 136 underwent thulium laser enucleation of the prostate. The average postoperative follow-up period was 30.2 months; during this period, 23 patients met the indication for cystoscopy, and all 23 were found to have urethral strictures upon cystoscopy examination. The median time from surgery to the discovery of stricture was approximately 4 months. Table 1 presents the distribution of urethral stricture locations among the patients. The membranous urethra was the most common site of urethral strictures, accounting for 69.2% of the cases, followed by the penile urethra (15.4%), bladder neck (11.5%), and the urethra navicular fossa (3.8%). Table 2 presents the results of a comparative analysis of preoperative characteristics between patients with and without urethral strictures. Notably, patients with urethral strictures had a significantly smaller prostate volume and transitional zone volume (*p* = 0.025) and a significantly higher Qmax than those without urethral strictures (10.6 vs. 8.4 mL/s, *p* = 0.010). Table 3 lists the perioperative outcomes. The weight of the enucleated specimen was significantly lower in patients with urethral strictures than in those without urethral strictures (11.4 vs. 20.9 g, *p* < 0.001). Additionally, patients with urethral strictures had a higher trial without catheter (TWOC) failure rate (34.8% vs. 3.1%, *p* < 0.001) and a higher incidence of postoperative UTIs (65.2% vs. 24.7%, *p* < 0.001). Patients with and without urethral strictures did not differ significantly in age, creatinine levels, prostate-specific antigen levels, PVR, IPSS, comorbidities, hospital stay duration, enucleated ratio, operating time, or choice of operating method. Table 4 presents the results of both univariate and multivariate logistic regression analyses that were conducted to determine the independent factors associated with the outcome. The parameters analyzed included prostate volume, T-zone volume, Qmax, specimen weight, TWOC failure, and postoperative UTI. The multivariate analysis results indicated that specimen weight (*p* = 0.036) and postoperative UTI (*p* = 0.001*)* were significantly associated with urethral stricture. TWOC failure was also significantly associated with urethral stricture, although the corresponding confidence interval was wider (odds ratio: 9.102 [2.514 to 32.947], *p* = 0.001). All 23 patients with urethral strictures had undergone cold-knife optical internal urethrotomy (OIU). Figure 1 illustrates the Qmax and PVR results obtained before and after the incision. On average, after the OIU, the patients’ Qmax increased significantly (6.6 mL/s), and their PVR decreased significantly (by 46.9 mL). Thus, the quality of urination improved significantly after the procedure. Of the 23 patients, 21 underwent a single OIU procedure, with an average time to stricture onset of 4 months. Only two patients developed recurrent strictures requiring multiple procedures (one underwent another surgery 3 months after the initial OIU, and the other underwent additional OIU at 6 months and 12 months). Both patients had experienced UTI within 1 month following the enucleation surgery.

## 4. Discussion

Iatrogenic injury is a leading cause of urethral strictures in well-resourced countries, contributing to 32% to 79% of all cases [5]. According to the literature, transurethral prostate surgery is the most common iatrogenic cause of urethral strictures, affecting 4.5% to 13% of patients and accounting for 41% of all cases [5]. The membranous urethra is the most common site of urethral strictures [5]. Numerous studies have focused on identifying risk factors for urethral strictures and bladder neck contracture (BNC) resulting from endoscopic prostate surgery for bladder outlet obstruction. A study involving 373 patients with a median follow-up period of 29.3 months explored risk factors for urethral strictures and BNC following TURP. The study demonstrated that a lower resection speed, urethral mucosa rupture, and continuous infection were linked to a higher risk of urethral strictures, and a higher storage score and smaller prostate size were associated with an increased risk of BNC [15]. Another retrospective study reported that the incidence of BNC was consistent across different surgical techniques (TURP, thulium vaporesection, and thulium laser enucleation) and that low prostate volume, recatheterization, and presence of two or more comorbidities were positively correlated with the development of BNC after surgery [16]. Furthermore, a systematic review and meta-analysis revealed that the overall incidence of urethral strictures was relatively low following endoscopic procedures for treating BPH. The highest incidence rate of 3.8% was observed after monopolar TURP, whereas the incidence rate following bipolar TURP was 2.1%. The lowest incidence rate of urethral strictures, at 1.7%, was observed after enucleation procedures [17]. However, the literature on this topic may have some minor methodological limitations. For example, the diameter of the resection scope sheath used in endoscopic surgery, the sources of cutting energy, the surgical techniques used by physicians, and the time points for follow-up vary across studies, all of which contribute to the inconsistent results reported by these studies. Furthermore, the definitions of iatrogenic trauma during prostate surgery, postoperative UTIs, and urethral strictures are not standardized, which may introduce potential biases. Therefore, we designed our follow-up protocol prospectively to maintain consistency across these variables to minimize bias. Patients were asked to attend follow-ups at 2 weeks, 3 months, and 6 months post-discharge for evaluation. Cystoscopy was proactively performed if symptoms worsened, and urodynamic test results deteriorated. Of our patients, 10.3% met the indication for cystoscopy, which is slightly higher than the proportion reported in the literature. All these patients were found to have urethral strictures during cystoscopy. Our study findings indicate that urethral strictures might be an underestimated complication associated with endoscopic prostate surgeries.

According to the literature, most patients develop urethral strictures within 6 months following transurethral surgery [18], and the stricture sites varied across studies [19]. In this study, the median time from surgery to the detection of stricture was approximately 4 months. The membranous urethra was the most common site, accounting for 69.2% of the cases. We speculate that most strictures in the membranous urethra may be associated with the surgical technique used during enucleation of the prostate. During the enucleation procedure, the resectoscope often serves as a fulcrum, utilizing leverage to separate the adenoma from the capsule [12,13]. The membranous urethra frequently becomes the point of maximal force application, which may lead to repeated compression and friction, potentially causing damage to the urothelial tissue and subsequently resulting in the formation of strictures.

In our study, patients with urethral strictures had smaller prostate volumes and smaller T zones prior to surgery. The weight of the enucleated specimen was also lower among these patients. Furthermore, multivariate analysis revealed that a smaller prostate resection weight and recatheterization were risk factors for urethral strictures. Our findings are consistent with those of Chen et al. [16]. Similar to their conclusions, our findings reveal that the energy source used during surgery was not a risk factor for urethral strictures. The reason why smaller prostates are more likely to lead to urethral strictures after endoscopic surgery is not thoroughly discussed in the literature. We hypothesize that in smaller prostates, the urethra and surrounding structures are more closely packed [20], making surgical navigation and tissue handling more difficult. The increased manipulation within a confined space can cause greater trauma and result in higher rates of postoperative fibrosis and scarring, which contribute to the development of strictures [20]. Moreover, prostatic blood flow was reported to be inversely correlated with prostate volume [21], and the blood supply to the healing tissues in a smaller prostate may be more easily compromised, leading to impaired healing and increased scarring. Meanwhile, in prostate enucleation surgery, smaller prostate volumes often result in smaller enucleated specimens. In our study, the logistic regression analysis also demonstrated that a smaller weight of the enucleated specimen is a risk factor for the development of urethral strictures, as shown in Table 4.

The occurrence of postoperative UTIs is another independent factor contributing to postoperative urethral strictures. In our study, 65.2% of patients with urethral strictures contracted a postoperative UTI. Urethral fibrosis may arise from intrinsic conditions but is often a result of urothelial damage caused by infection [22]. TWOC failure was another significant risk factor for urethral strictures (odds ratio: 7.44). Patients with TWOC failure experienced postoperative urinary retention and repeated catheter insertions, both of which could potentially damage the urethral epithelium. The normal urethra is lined by pseudostratified columnar epithelium, which is anchored to a basement membrane. Beneath this membrane lies connective tissue composed of fibroblasts within an extracellular matrix comprising collagen, proteoglycans, elastic fibers, and glycoproteins [23]. Below this layer is the spongiosum, consisting of vascular sinusoids and smooth muscle. After urethral infection or trauma, a pathological change occurs, in which the normal epithelium is replaced by squamous metaplasia, which subsequently leads to fibrosis and the development of strictures [23].

This study has several limitations. First, the sample size (246 patients) was relatively small, which may limit the generalizability of the findings, considering the complexity of urethral strictures and the variability in their incidence across patients. Second, this study was conducted in a single hospital by one surgeon, which further restricts the applicability of these results to other settings owing to potential variations in surgical outcomes. Finally, despite an average postoperative follow-up period of 30.2 months, the active intervention and assessment period for patients was only limited to 6 months. Our results indicate that the average time to the onset of stricture was 4 months, but this short follow-up duration may have overlooked the development of late strictures. Nevertheless, we still believe that this study holds significant practical value because it is the first to investigate the incidence of urethral strictures following endoscopic enucleation of the prostate.

## 5. Conclusions

Our study revealed that urethral strictures constitute a prominent and possibly underestimated complication after endoscopic enucleation of the prostate, with the membranous urethra being the most affected site. The findings suggest that smaller prostate size, lower resected tissue weight, TWOC failure, and postoperative UTIs are significant risk factors for the development of strictures. Our study highlights the importance of careful surgical techniques and diligent postoperative monitoring to minimize the incidence of urethral strictures.

## Figures and Tables

**Table 1:** Location of the urethral stricture.

Location	N (%)
Membranous urethra	18 (69.2%)
Penile urethra	4 (15.4%)
Bladder neck	3 (11.5%)
Navicular fossa of urethra	1 (3.8%)

**Table 2:** Preoperative demographic characteristics.

Parameter (Mean ± SD, %)	Stricture (*n* = 23)	No Stricture (*n* = 223)	*p* Value
Age (years)	64.8 ± 8.8	68.5 ± 8.9	0.058
Prostate volume (cc)	43.5 ± 16.6	53.5 ± 20.8	0.025 *
T zone (cc)	18.2 ± 13.4	25.3± 14.5	0.025 *
Creatinine (mg/dL)	0.96 ± 0.32	0.99 ± 0.55	0.743
PSA (ng/mL)	6.0 ± 7.4	6.2 ± 8.2	0.705
Qmax (mL/s)	10.6 ± 3.3	8.4 ± 3.7	0.010 *
PVR (mL)	87.1 ± 70.4	99.5 ± 131.1	0.669
IPSS_v	15.4 ± 3.2	15.0 ± 3.4	0.591
IPSS_s	8.4 ± 3.4	9.2 ± 3.5	0.294
IPSS_t	23.9 ± 4.2	24.3 ± 4.7	0.691
DM (%)	26	22	0.615
HTN (%)	52	50	0.827
CAD (%)	4	12	0.265
UR (%)	17	14	0.648

Abbreviations: SD: standard deviation; T: transitional; PSA: prostate-specific antigen; Qmax: maximum urinary flow rate; PVR: post-void residual volume; IPSS: International Prostate Symptom Score; DM: diabetes mellitus; HTN: hypertension; CAD: coronary arterial disease; UR: urinary retention. *: statistically significant difference

**Table 3:** Perioperative demographic characteristics.

Parameter (Mean ± SD, %)	Stricture (*n* = 23)	No Stricture (*n* = 223)	*p* Value
Hospital stay (days)	2.2 ± 0.5	2.2 ± 0.7	0.797
Weight of specimen (g)	11.4 ± 7.3	20.9 ± 14.3	<0.001
Enucleated ratio (%)	73.5%	88.5%	0.171
Operation time (min)	69.7 ± 25.0	83.1 ± 32.3	0.055
Operation methods (B-TUEP/ThuLEP)	7/16 (43.8%)	102/121 (84.3%)	0.159
TWOC fail (*n*, %)	8 (34.8%)	7 (3.1%)	<0.001
Post-op UTI (*n*, %)	15 (65.2%)	55 (24.7%)	<0.001

Abbreviations: SD: standard deviation; B-TUEP: bipolar transurethral enucleation of the prostate; ThuLEP: thulium laser enucleation of the prostate; Post-op: postoperative; TWOC: trial without catheter; UTI: urinary tract infection.

**Table 4:** Logistic regression analysis.

Parameter	Univariate OR (95%CI), *p*	Multivariate OR (95%CI), *p*
Prostate volume	0.966 (0.937–0.996), *p* = 0.028	1.001 (0.929–1.079), *p* = 0.978
T zone	0.952 (0.911–0.994), *p* = 0.027	1.002 (0.926–1.129), *p* = 0.662
Qmax	1.150 (1.029–1.286), *p* = 0.014	1.065 (0.922–1.230), *p* = 0.392
Weight of specimen (g)	0.915 (0.863–0.970), *p* = 0.003	0.886 (0.792–0.992), *p* = 0.036 *
TWOC fail	16.457 (5.256–51.531), *p* < 0.001	7.440 (1.033–53.598), *p* = 0.046 *
Post-op UTI	5.727 (2.304–14.235), *p* < 0.001	9.102 (2.514–32.947), *p* = 0.001 *

Abbreviations: OR: odds ratio; CI: confidence interval; Qmax: maximum urinary flow rate; TWOC: trial without catheter; UTI: urinary tract infection. *: statistically significant difference

## Data Availability

The data used to support the findings of this study are available from the corresponding author upon request.
